# The effect of changes in cerebral blood flow on cognitive function during exercise

**DOI:** 10.14814/phy2.12163

**Published:** 2014-09-28

**Authors:** Shigehiko Ogoh, Hayato Tsukamoto, Ai Hirasawa, Hiroshi Hasegawa, Norikazu Hirose, Takeshi Hashimoto

**Affiliations:** 1 Graduate School of Engineering, Toyo University, Kawagoe‐Shi, Saitama, Japan; 2 Graduate School of Sport and Health Science, Ritsumeikan University, Shiga, Japan; 3 Graduate School of Integrated Arts and Sciences, Hiroshima University, Hiroshima, Japan; 4 School of Sport Sciences, Waseda University, Saitama, Japan

**Keywords:** arterial blood pressure, brain, cognition, humans, hypercapnia

## Abstract

No studies have identified the direct effect of changes in cerebral blood flow (CBF) on cognitive function at rest and during exercise. In this study, we manipulated CBF using hypercapnic gas to examine whether an increase in CBF improves cognitive function during prolonged exercise. The speed and the accuracy of cognitive function were assessed using the Stroop color‐word test. After the Stroop test at rest, the subjects began exercising on a cycling ergometer in which the workload was increased by 0.5 kilopond every minute until a target heart rate of 140 beats/min was achieved. Then, the subjects continued to cycle at a constant rate for 50 min. At four time points during the exercise (0, 10, 20, 50 min), the subjects performed a Stroop test with and without hypercapnic respiratory gas (2.0% CO_2_), with a random order of the exposures in the two tests. Despite a decrease in the mean blood flow velocity in the middle cerebral artery (MCA *V*_mean_), the reaction time for the Stroop test gradually decreased during the prolonged exercise without any loss of performance accuracy. In addition, the hypercapnia‐induced increase in MCA *V*_mean_ produced neither changes in the reaction time nor error in the Stroop test during exercise. These findings suggest that the changes in CBF are unlikely to affect cognitive function during prolonged exercise. Thus, we conclude that improved cognitive function may be due to cerebral neural activation associated with exercise rather than global cerebral circulatory condition.

## Abstract (precis)

e12163

We found that even prolonged exercise improved cognitive function, which was unlikely to have been affected by changes in cerebral blood flow. Exercise‐induced improvements in cognitive function may be due to cerebral neural activation associated with exercise rather than cerebral circulatory condition. The finding would advance our understanding of physiological mechanisms underlying the regulation of the cognitive function.

## Introduction

Aging is associated with a progressive decline in cerebral blood flow (CBF) (Ainslie et al. 2008; Bertsch et al. 2009), and it has been suggested that this age‐related decline in resting CBF is associated with poor cognitive function (Bertsch et al. 2009). In addition, Marshall et al. (2001) reported that the transient occlusion of CBF in patients with cardiovascular disease reversibly impaired cognition. These previous findings indicate that cognitive function may be modified by changes in CBF. Importantly, habitual physical exercise may offset some of the ‘normal’ age‐associated process of global cerebral atrophy, suggesting that active living might induce an increase in global CBF that might delay the cerebrovascular‐related brain disease that occurs with aging (Ainslie et al. 2008).

During dynamic exercise at mild‐to‐moderate intensity, increases in cerebral metabolism are paralleled by transient increases in internal carotid artery (ICA) blood flow (Hellstrom et al. 1996; Sato et al. 2011) and middle cerebral artery (MCA) mean blood flow velocity (MCA *V*_mean_) (Ogoh et al. 2005a,c; Ogoh 2008; Ogoh and Ainslie 2009a,b). This increase in CBF may be required to meet the metabolic demands of cerebral neuronal activity during dynamic exercise (Ide and Secher 2000; Ogoh 2008; Ogoh and Ainslie 2009a,b). Interestingly, cognitive function improves during a single bout of moderate exercise (Brisswalter et al. 2002; McMorris et al. 2011; Lucas et al. 2012). In contrast, during prolonged dynamic exercise, CBF gradually decreases toward resting values in association with hyperventilation (Ogoh et al. 2005b). Similar to the decrease in CBF, the exercise‐induced facilitation of cognitive function disappears during such prolonged exercise (Grego et al. 2005). Taken together, these results suggest that exercise‐induced transient changes in CBF may alter cognitive function. However, no previous studies have manipulated CBF to examine whether acute changes in CBF affect cognitive function at rest and during exercise. We hypothesize that cognitive function is impaired during prolonged exercise and can be restored by an increase in CBF. In this study, we increased CBF using hypercapnic respiratory gas to examine the effect of a change in CBF on cognitive function during prolonged exercise.

## Methods

### Subjects

Seven healthy young men (mean ± SEM; age = 20.4 ±0.6 years, height = 169.9 ± 1.5 cm, weight = 66.2 ± 2.2 kg) volunteered for this study. Before the study, each subject provided written, informed consent after all potential risks and procedures were explained. To ensure that the subjects would be able to perform the full hour of dynamic exercise required by the protocol, most of the recruited subjects were men who routinely participated in high‐intensity exercise training 5 days a week (e.g., cross‐country ski players, lacrosse players). All experimental procedures and protocols were approved by the ethics committee of Waseda University (IRB No. 2013‐114) and conformed to the standards set by the Declaration of Helsinki. The subjects were free of any known cardiovascular and pulmonary disorders and were not using any prescribed or over‐the‐counter medications. The subjects were asked to abstain from caffeinated beverages for 12 h, strenuous physical activity and alcohol for at least 24 h, and from any food and drink except for water for 4 h before the experiment.

### Experimental protocol

**Figure 1.**
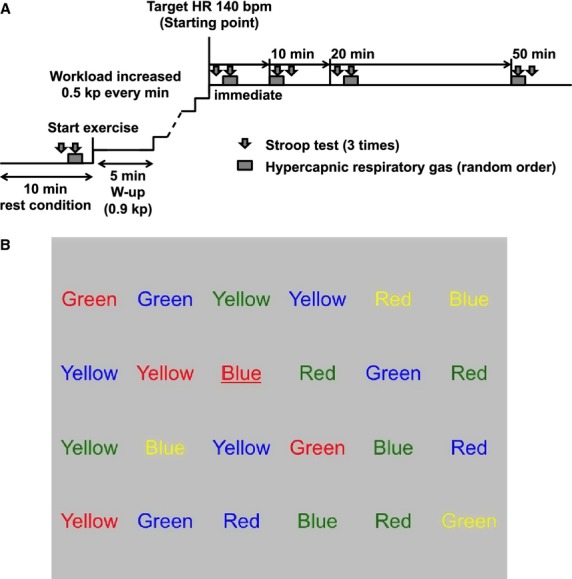
(A) Experimental protocol. (B) An example of randomized incongruent words and colors‐recognition tests. The example indicates that the word is blue (underlined) while it is printed in red, and then the subjects have to press “blue” key and not “red” key.

Before the experiment, each subject visited the laboratory for familiarization with the experimental protocol and equipment and completed a series of practice trials of the cognitive tasks to reduce learning effects. On the day of the experiment, each subject performed a cognitive task (Stroop color‐word test) during prolonged cycling exercise on a bike ergometer (PowerMaxVIII, Konami co. Ltd., Tokyo, Japan). Figure 1 summarizes the experimental protocol. Prior to the day of the experiment, subjects were trained to perform Stroop color‐word test until they achieved a constant score before the day of the experiment. The test was repeated 15 times (15 trials: one trial of a Stroop test presented 24 stimulus words as mentioned below) to rapidly perform this task before the experiment to avoid any learning effect. Following the application of the instrumentation, baseline recording was performed for 10 min while the subject was at rest. Each subject then performed the cognitive task while sitting on the bike ergometer under the prescribed conditions (with and without inhalation of 2.0% CO_2_, a hypercapnic respiratory gas). To test our hypothesis, subjects performed prolonged exercise (50 min) without full exertion, and CBF was monitored to determine if a gradual decrease occurred via hyperventilation near the end of the prolonged exercise period. After the pre‐exercise tests of cognitive function, the subjects began cycling exercise on the bike ergometer at 0.9 kilopond (kp) for 5 min as a warm‐up exercise. The exercise workload was subsequently increased by 0.5 kp every minute until the subject's heart rate reached the target of 140 beats/min, that is, 178 ± 28 W (mean ± SE). After reaching this heart rate, the subjects continued to cycle at a constant rate for 50 min. At each of the examined time points during the exercise (0 min [immediate], 10 min, 20 min, and 50 min after starting to exercise), the subjects performed the cognitive task both with (Hypercapnia) and without (Control) the hypercapnic respiratory gas. This hypercapnic‐stimulation (Hypercapnia) was randomized (Fig. 1A). Briefly, the subjects breathed through a face mask and performed two randomly assigned respiratory interventions: (1) normocapnia (room air) for the moment of the Stroop test, and (2) mild hypercapnia (2% CO_2_ in 21% O_2_ and N_2_ balance from a 200‐liter Douglas bag) for the moment of the Stroop test. Stroop test was started 30 sec after the normocapnic or hypercapnic stimulation. The hypercapnic stimulation was terminated immediately after the end of the each Stroop test. All studies were performed at a constant temperature between 22 and 24°C.

### Cognitive function (Stroop task)

Cognitive function was assessed for both speed and accuracy using the Stroop color word test (Stroop 1935), which is a well‐known paradigm for investigating aspects of cognitive performance that depend on executive functioning; specifically, selective attention to specific information and the inhibition of prepotent response during decision‐making tasks involving stimuli and responses (MacLeod 1991). The Stroop test was administered on a computer screen using randomized incongruent words and colors‐recognition tests (modified using Excel VBA) in a temperature‐controlled, noise‐free environment to minimize distractions and increase the comfort level of the subject. The Stroop test presents the names of four colors (Red, Green, Yellow, and Blue) displayed in incongruent colors (e.g., Green, Yellow, Blue, and Red, respectively) on the computer screen (Fig. 1B). We prepared a color‐labeled tenkeyboard: number 1 key was labeled green, number 2 key was labeled yellow, number 3 key was labeled red, and number 4 key was labeled blue. The subjects were required to press the color‐labeled key that corresponds to the text meaning of the stimulus word as quickly and accurately as possible. One trial of a Stroop test presented 24 stimulus words (Fig. 1B), and at each time point, each subject repeated three test trials, between which there was a couple second time delay for the test program reset and restart. We measured the average of the reaction times and the number of errors in the responses for three test trials to assess the speed and accuracy, respectively, at each time point during the exercise, as well as at rest.

### Measurements

The heart rates (HR) were monitored using a lead II electrocardiogram. The blood flow velocity in the left MCA was determined by transcranial Doppler ultrasonography (Multidop T; DWL, Sipplingen, Germany). The expired air was sampled breath‐by‐breath, and minute ventilation (V_E_) and the end‐tidal partial pressure of carbon dioxide (P_ET_CO_2_) were measured using a gas analyzer system (AE‐310S, Minato Medical Science Co., Osaka, Japan).

### Statistics

The statistical analyses were conducted using SigmaStat software (Jandel Scientific Software, SPSS Inc., Chicago, IL). The data were subsequently analyzed using a one‐way repeated measures analysis of variance (ANOVA) test and post hoc Student's Newman–Keul tests. Hypercapnia‐induced changes were evaluated by comparing Control and Hypercapnia using a paired Student's *t*‐test. The data are expressed as the mean ± SEM, and significance was set at *P* < 0.05.

## Results

### Hemodynamic response to prolonged exercise

All subjects performed prolonged exercise (50 min) without full exertion. Immediately after the beginning of exercise, HR, V_E_, and P_ET_CO_2_ increased from resting levels (Table 1). The increase in V_E_ was well maintained throughout the exercise period, although the HR and percentage of predicted maximal HR (% HR _max_) gradually increased until the 50‐min time point. P_ET_CO_2_ gradually decreased throughout the exercise period and returned to the resting level at 50 min.

**Table 1.** Cardiovascular and respiratory variables at rest and during prolonged exercise under control or hypercapnia.

	Rest	Immediate	10 min	20 min	50 min
Control					
HR, bpm	72 ± 5	142 ± 6^**^	149 ± 6^**^^,^^†^^,^^##^	153 ± 5^**^^,^^††^^,^^##^	168 ± 5^**^^,^^††^
(% HRmax)		(69.0 ± 2.6)	(74.6 ± 2.7)^**^	(76.2 ± 2.3)^**^^,^^†^	(84.1 ± 2.3)^**^^,^^†^^,^^#^
V_E_, L/min	18 ± 2	96 ± 11^**^	102 ± 9^**^	100 ± 7^**^	107 ± 8^**^
P_ET_CO_2_, mmHg	40 ± 2	49 ± 2^*^	46 ± 2^*^^,^^#^	46 ± 2^*^^,^^##^	44 ± 2^†^
Hypercapnia					
HR, bpm	71 ± 5	142 ± 4^**^	149 ± 6^**^^,^^††^^,^^##^	154 ± 5^**^^,^^††^^,^^‡^^,^^#^	168 ± 5^**^^,^^††^
V_E_, l/min	20 ± 1	84 ± 10^**^	98 ± 11^**^	113 ± 16^**^	114 ± 8^**^^,^^††^
P_ET_CO_2_, mmHg	46 ± 1^$^	56 ± 2^**^^,^^$^	54 ± 3^*^^,^^$^	53 ± 2^**^^,^^†^^,^^#^^,^^$^	52 ± 2^**^^,^^††^^,^^$^

*P *< 0.05, **P < 0.01 vs. rest; ^†^*P *< 0.05, ^††^*P *< 0.01 vs. immediate; ^‡^*P *< 0.05 vs. 10 min; ^#^*P *< 0.05, ^##^*P *< 0.01 vs. 50 min; ^$^*P *< 0.01 vs. Control.

### The effect of CBF on cognitive function during prolonged exercise

**Figure 2.**
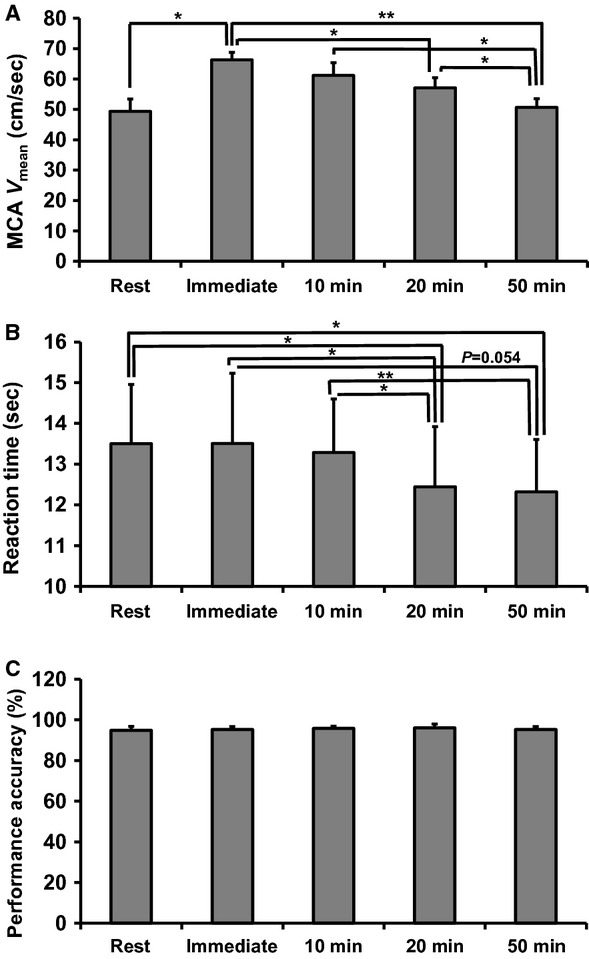
Cognitive function improved during prolonged exercise despite decreases in CBF. (A) MCA mean blood flow velocity (MCA *V*_mean_), (B) reaction time, (C) performance accuracy at rest, and during prolonged exercise. The values shown represent the mean ± SEM. *P < 0.05, **P < 0.01 between the groups.

During prolonged exercise, the MCA *V*_mean_ gradually decreased: MCA *V*_mean_ at the 20 min time point was significantly decreased compared to immediately after the subjects started to exercise (*P* < 0.05), and MCA *V*_mean_ at 50 min was significantly decreased compared to immediately after the subjects started to exercise (*P* < 0.01), at 10 min (*P* < 0.05), and at 20 min (*P* < 0.05) (Fig. 2A). Despite a decrease in MCA *V*_mean_, the reaction time observed on the Stroop test gradually decreased during exercise: the reaction time at 20 min was significantly less than the resting value (*P* < 0.05), immediately after the subjects started to exercise (*P* < 0.05), and at 10 min (*P* < 0.05) (Fig. 2B). The reaction time at 50 min was significantly less than the resting level (*P* < 0.05) and at 10 min (*P* < 0.01).The performance accuracy measured by the Stroop test was unchanged throughout the exercise period (Fig. 2C).

### The effect of hypercapnia‐induced increase in CBF on cognitive function during prolonged exercise

**Figure 3.**
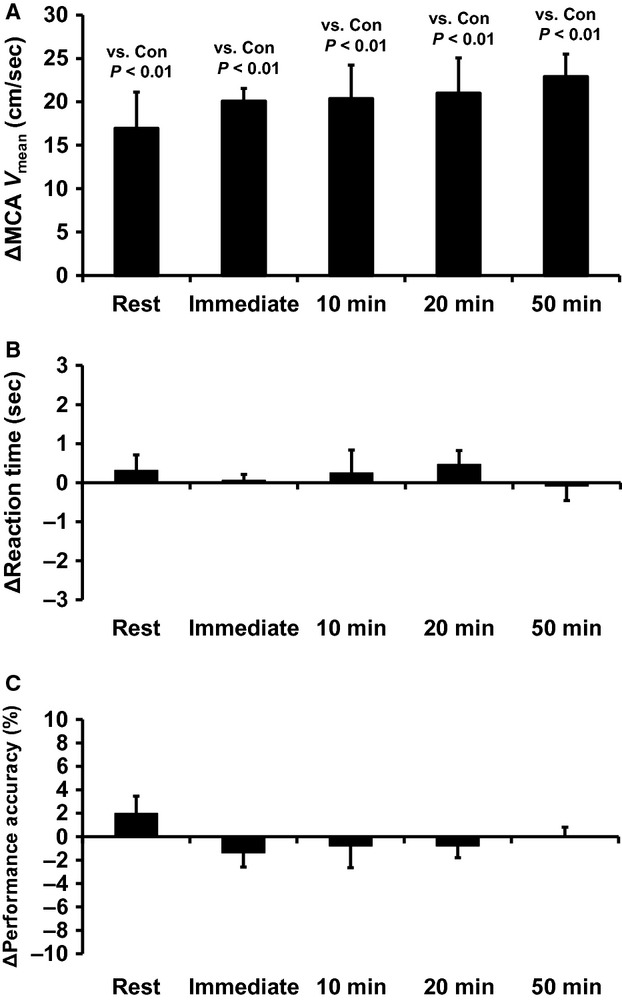
Hypercapnia‐induced increase in CBF did not affect cognitive function at rest as well as any time point during the prolonged exercise. (A) Changes in MCA mean blood flow velocity (ΔMCA *V*_mean_) in response to hypercapnic stimulation at rest and during exercise. Hypercapnic stimulation significantly increased the MCA *V*_mean_ at each time point (*P *< 0.01). Changes in (B) reaction time (Δreaction time), (C) performance accuracy (Δperformance accuracy) in response to hypercapnic stimulation at rest and during exercise. Hypercapnic stimulation did not change reaction time and performance accuracy. The values shown represent the mean ± SEM.

Hypercapnic stimulation quickly and significantly increased the P_ET_CO_2_ (*P* < 0.01 vs. Control, Table 1) and hence quickly and significantly increased the MCA *V*_mean_. Importantly, the hypercapnic stimulation did not alter HR. The reaction time for the Stroop test was not significantly different to the Control at any time point measured (Fig. 3A, and Fig. 3B), whereas hypercapnic stimulation did not change the performance accuracy measured by the Stroop test (Fig. 3C).

## Discussion

The main finding of this study was that cognitive function improved during prolonged exercise despite decreases in CBF. In addition, a hypercapnia‐induced increase in CBF did not affect cognitive function at rest or at any time point during the prolonged exercise. These findings suggest that improved cognitive function during exercise may be due to the cerebral neural activation associated with exercise, rather than the cerebral perfusion.

This study is the first to examine the effect of changes in cerebral perfusion on cognitive function at rest and during exercise. Previous studies have demonstrated that age‐related decline in resting CBF is associated with poorer cognitive function (Bertsch et al. 2009). In addition, Marshall et al. (2001) reported that the transient occlusion of CBF in patients with cerebrovascular disease reversibly impaired cognition (attention). These studies have suggested that changes in cognitive function may be due to alteration in cerebral perfusion. However, no studies have previously investigated the effect of an isolated change in CBF on cognitive function in humans by manipulating CBF. Recently, Lucas et al. (2012) measured both CBF and cognitive function during a single bout of exercise and provided evidence of a significant relationship between these parameters, particularly at rest. However, they suggested that the relationship between CBF and cognitive function becomes uncoupled during exercise. Our results support their suggestion. However, the study by Lucas et al. (2012) could not identify the precise relationship, because CBF changes were not isolated from the physiological responses to exercise. Exercise does not change only the CBF, but also other physiological factors which may affect cognitive function. Thus, the results of Lucas' study regarding cognitive function may include confounding factors. In this study, despite a decrease in MCA *V*_mean_, cognitive function was improved during prolonged exercise. In addition, we isolated the effect of CBF on cognitive function during exercise by manipulating CBF with hypercapnic stimulation to independently increase CBF (e.g., no alteration of HR under the hypercapnic condition as compared to Control) and found that a hypercapnia‐induced increase in MCA *V*_mean_ did not alter the cognitive function at any time point during the exercise. These findings clearly demonstrated that a change in CBF does not affect cognitive function at rest or during exercise and that a change in cognitive function during exercise, which was observed in previous studies, is due to other mechanisms.

The concept that cognitive function is associated with CBF is based on the facts that a change in CBF reflects oxygen delivery to the brain and that hypoxia has the potential to impair brain function (Hornbein 2001). In this study, the HR and relative intensity (% HR_max_) gradually increased during exercise despite an applying constant workload (i.e., cardiovascular drift). This cardiovascular drift, which was corresponding to the previous studies (Fritzsche et al. 1999; Ogoh et al. 2005b), was supposed to be caused by progressive increases in cutaneous blood flow and venous volume as body temperature rises, and also increases in whole body sympathetic activity (Fritzsche et al. 1999; Wingo et al. 2005). More importantly, this cardiovascular drift might result in increasing cerebral neural activity or metabolism, indicating that the prolonged exercise indeed increases demand of oxygen in the brain. Furthermore, in this study, the prolonged exercise gradually decreased CBF. However, cognitive function was not impaired throughout the prolonged exercise despite a conflict between an increase in cerebral metabolism and a decrease in CBF. In addition, hypercapnia‐induced increases in CBF did not improve cognitive function. Taken together, for cognitive function, an alteration in CBF may not be paralleled by oxygen delivery to the brain or by increased cerebral metabolism.

Previous studies have demonstrated that during heavy exercise, compensatory increases in the uptake (a–v difference) of lactate, glucose and oxygen support elevated brain neural activity and metabolism (Ide and Secher 2000). Furthermore, normobaric hypoxia did not impair cognitive function, despite a significant decrease in cerebral oxygenation during exercise (Ando et al. 2013). In this study, at least during exercise, it appears that global CBF was enough to satisfy metabolic demand to perform exercise. Given that increases in brain neuronal activity and metabolism do not parallel increases in CBF (Miyazawa et al. 2012), cognitive function may be affected by the extensive activation of motor and sensory systems with the high‐order function of the prefrontal cortex rather than by cerebral perfusion during prolonged exercise.

In addition to this study, it has been reported that exercise results in a mild enhancement of cognitive function (Colcombe and Kramer 2003; Tomporowski 2003), which may be associated with exercise workload. Mild and moderate exercise improves cognitive function, whereas heavy exercise is unlikely to improve function. McMorris et al. (2011) reported that an acute, intermediate level of exercise has a strong beneficial effect on the response speed, but not on its accuracy, in a working memory task. Brisswalter et al. (2002) also reported that decisional performance was improved immediately after the adrenaline threshold during incremental exercise. They suggested that this improvement may be enhanced by nutritional factors, but did not seem to be influenced by the level of fitness. On the other hand, Tomporowski et al. reported differences in cognitive function during and after acute exercise between different modes of exercise (Tomporowski 2003). This phenomenon could be explained by changes in CBF, because heavy exercise does not increase CBF. However, our data demonstrated that cognitive function was not affected by changes in CBF during exercise. Why is cognitive function modified by exercise? Endo et al. (2013) reported that dynamic exercise at a moderate intensity might improve cognitive function through increased prefrontal oxygenation. These previous studies evaluated cognitive function at least 5–15 min after the exercise bout had ceased. However, the pattern of neural activation in the brain associated with a particular task rapidly returns to baseline levels after the cessation of that task. In other words, a delay of even a few minutes would be sufficient to normalize any exercise‐induced change in neural activity (Dietrich and Sparling 2004), and cognitive function measured after exercise may be the result of other possible physiological confounding factors. Indeed, Vasques et al. reported differences in the cognitive function between, during, and after physical exercise in depressed elderly persons (Vasques et al. 2011). These previous studies did not identify the direct effect of exercise on cognitive function, at least with respect to neural activity. Therefore, the mechanism of change in cognitive function during excise should be further elucidated.

It is widely accepted that the exercise–cognition interaction (dual tasks) is complex. Dietrich and Sparling (Dietrich and Sparling 2004) reported an impaired performance of a complex task that depends on frontal functions. They proposed the transient hypofrontality hypothesis, which suggests that cognitive functions associated with the frontal areas are impaired during acute exercise because the brain prioritizes motor control and the maintenance of vital function (e.g., blood pressure and temperature control). These results, which are contradictory to our findings, may be due to differences in the cognitive tasks studied. The type of cognitive task may be an important factor affecting experimental results (Etnier and Chang 2009; Lambourne and Tomporowski 2010). In addition, acute moderate exercise differentially affects some specific aspects of cognitive function (Davranche and McMorris 2009). Davranche and McMorris (Davranche and McMorris 2009) showed that some cognitive functions are impaired (e.g., selective inhibition, response inhibition), whereas others (e.g., top‐down cognition control, reaction time) are not altered, and in contrast, were fully efficient. In this study, cognitive function was assessed for both speed and accuracy using a Stroop task (Stroop 1935). This task may be simple enough to be positively affected by arousal. However, for tasks with increased difficulty (Dietrich and Sparling 2004), such as the Paced Auditory Additional Task, Peabody Picture Vocabulary Test, the Brief Kaufman Intelligence Test and the Wisconsin Card Sorting Task, arousal is not likely to lead to improved performance.

### Limitations

This study has some limitations that should be considered. We assessed a small number of subjects. More importantly, we assessed well‐trained athletes for the protocol of this study. Thus, subjects of average fitness or older subjects with reduced cerebral perfusion may provide different results. However, in this protocol, the subjects needed to be able to cycle at high intensity for 1 h. Subjects with low and average fitness levels may make an effort to continue heavy exercise for 1 h, and this effort may affect cognitive function. Although we did not measure arousal level, relatively moderate excise for the subjects in this study might affect arousal level, and hence affect cognitive function (Kamijo et al. 2004; Murray and Russoniello 2012). In addition, Stroop interference should have adopted in this study to advance our understanding of the effect of a prolonged exercise on executive function, a specifically defined cognitive process (Byun et al. 2014). Second, a potential limitation of the transcranial Doppler ultrasonography measurement should be noted. Vasoconstriction of the insonated vessel increases MCA *V* at any given volume of flow. However, in humans, the MCA diameter appears to remain relatively constant under a variety of conditions (Schreiber et al. 2000; Serrador et al. 2000). Thus, we would argue that the beat‐to‐beat changes in MCA *V* reflected changes in flow (Ogoh et al. 2005a,b,c). Another limitation is that exercise may cause noise in the acquisition of the MCA *V* signal, but a visual inspection of the MCA *V* waveforms suggested that this was not the case in our study (Ogoh et al. 2005c). Finally, as we used the target HR to determine exercise workload, a different fitness does not change the relative intensity at least at the beginning of this protocol. However, changes in relative intensity (HR drift) during exercise should be different between individuals albeit small variability, and might affect neural activity. On the other hand, hypercapnia has been shown to reduce neural activation (Zappe et al. 2008); thus, it cannot be ruled out that the effect of the increase in CBF on cognitive function may be attenuated by hypercapnia induced this phenomenon.

## Conclusions

We examined whether acute changes in CBF directly affect cognitive function at rest and during exercise. To test this simple question, we manipulated CBF using hypercapnic respiratory gas during at rest and exercise. During prolonged exercise, cognitive function was improved, despite a decrease in MCA *V*_mean_. In addition, an acute increase in MCA *V*_mean_ by hypercapnic stimulation did not affect cognitive function throughout prolonged exercise. Therefore, exercise‐induced changes in CBF do not alter cerebral metabolism to preserve cognitive performance. However, we cannot rule out the possibility that neural activity, for example, central fatigue, modifies cognitive function.

## Acknowledgments

The authors appreciate the time and effort expended by the volunteer subjects. We are also grateful to Sadayoshi Sakai for the experimental support.

## Conflict of Interest

The authors declare no conflict of interest.

## References

[b1] Ainslie P. N. Cotter J. D. George K. P. Lucas S. Murrell C. Shave R. 2008 Elevation in cerebral blood flow velocity with aerobic fitness throughout healthy human ageing. J. Physiol.; 586:4005-4010. 18635643 10.1113/jphysiol.2008.158279 PMC2538930

[b2] Ando S. Hatamoto Y. Sudo M. Kiyonaga A. Tanaka H. Higaki Y. 2013 The effects of exercise under hypoxia on cognitive function. PLoS ONE; 8:e63630 23675496 10.1371/journal.pone.0063630 PMC3651238

[b3] Bertsch K. Hagemann D. Hermes M. Walter C. Khan R. Naumann E. 2009 Resting cerebral blood flow, attention, and aging. Brain Res.; 1267:77-88. 19272361 10.1016/j.brainres.2009.02.053

[b4] Brisswalter J. Collardeau M. Rene A. 2002 Effects of acute physical exercise characteristics on cognitive performance. Sports Med.; 32:555-566. 12096929 10.2165/00007256-200232090-00002

[b5] Byun K. Hyodo K. Suwabe K. Ochi G. Sakairi Y. Kato M. 2014 Positive effect of acute mild exercise on executive function via arousal‐related prefrontal activations: an fNIRS study. Neuroimage; 98:336-345. 24799137 10.1016/j.neuroimage.2014.04.067

[b6] Colcombe S. Kramer A. F. 2003 Fitness effects on the cognitive function of older adults: a meta‐analytic study. Psychol. Sci.; 14:125-130. 12661673 10.1111/1467-9280.t01-1-01430

[b7] Davranche K. McMorris T. 2009 Specific effects of acute moderate exercise on cognitive control. Brain Cogn.; 69:565-570. 19138814 10.1016/j.bandc.2008.12.001

[b8] Dietrich A. Sparling P. B. 2004 Endurance exercise selectively impairs prefrontal‐dependent cognition. Brain Cogn.; 55:516-524. 15223198 10.1016/j.bandc.2004.03.002

[b9] Endo K. Matsukawa K. Liang N. Nakatsuka C. Tsuchimochi H. Okamura H. 2013 Dynamic exercise improves cognitive function in association with increased prefrontal oxygenation. J. Physiol. Sci.; 63:287-298. 23661275 10.1007/s12576-013-0267-6 PMC10717244

[b10] Etnier J. L. Chang Y. K. 2009 The effect of physical activity on executive function: a brief commentary on definitions, measurement issues, and the current state of the literature. J. Sport Exerc. Psychol.; 31:469-483. 19842543 10.1123/jsep.31.4.469

[b11] Fritzsche R. G. Switzer T. W. Hodgkinson B. J. Coyle E. F. 1999 Stroke volume decline during prolonged exercise is influenced by the increase in heart rate. J. Appl. Physiol. (1985); 86:799-805. 10066688 10.1152/jappl.1999.86.3.799

[b12] Grego F. Vallier J. M. Collardeau M. Rousseu C. Cremieux J. Brisswalter J. 2005 Influence of exercise duration and hydration status on cognitive function during prolonged cycling exercise. Int. J. Sports Med.; 26:27-33. 15643531 10.1055/s-2004-817915

[b13] Hellstrom G. Fischer‐Colbrie W. Wahlgren N. G. Jogestrand T. 1996 Carotid artery blood flow and middle cerebral artery blood flow velocity during physical exercise. J. Appl. Physiol. (1985); 81:413-418. 8828693 10.1152/jappl.1996.81.1.413

[b14] Hornbein T. F. 2001 The high‐altitude brain. J. Exp. Biol.; 204:3129-3132. 11581326 10.1242/jeb.204.18.3129

[b15] Ide K. Secher N. H. 2000 Cerebral blood flow and metabolism during exercise. Prog. Neurobiol.; 61:397-414. 10727781 10.1016/s0301-0082(99)00057-x

[b16] Kamijo K. Nishihira Y. Hatta A. Kaneda T. Kida T. Higashiura T. 2004 Changes in arousal level by differential exercise intensity. Clin. Neurophysiol.; 115:2693-2698. 15546777 10.1016/j.clinph.2004.06.016

[b17] Lambourne K. Tomporowski P. 2010 The effect of exercise‐induced arousal on cognitive task performance: a meta‐regression analysis. Brain Res.; 1341:12-24. 20381468 10.1016/j.brainres.2010.03.091

[b18] Lucas S. J. Ainslie P. N. Murrell C. J. Thomas K. N. Franz E. A. Cotter J. D. 2012 Effect of age on exercise‐induced alterations in cognitive executive function: relationship to cerebral perfusion. Exp. Gerontol.; 47:541-551. 22230488 10.1016/j.exger.2011.12.002

[b19] MacLeod C. M. 1991 Half a century of research on the Stroop effect: an integrative review. Psychol. Bull.; 109:163-203. 2034749 10.1037/0033-2909.109.2.163

[b20] Marshall R. S. Lazar R. M. Pile‐Spellman J. Young W. L. Duong D. H. Joshi S. 2001 Recovery of brain function during induced cerebral hypoperfusion. Brain; 124:1208-1217. 11353736 10.1093/brain/124.6.1208

[b21] McMorris T. Sproule J. Turner A. Hale B. J. 2011 Acute, intermediate intensity exercise, and speed and accuracy in working memory tasks: a meta‐analytical comparison of effects. Physiol. Behav.; 102:421-428. 21163278 10.1016/j.physbeh.2010.12.007

[b22] Miyazawa T. Horiuchi M. Ichikawa D. Sato K. Tanaka N. Bailey D. M. 2012 Kinetics of exercise‐induced neural activation; interpretive dilemma of altered cerebral perfusion. Exp. Physiol.; 97:219-227. 22041980 10.1113/expphysiol.2011.061978

[b23] Murray N. P. Russoniello C. 2012 Acute physical activity on cognitive function: a heart rate variability examination. Appl. Psychophysiol. Biofeedback; 37:219-227. 22543813 10.1007/s10484-012-9196-z

[b24] Ogoh S. 2008 Autonomic control of cerebral circulation: exercise. Med. Sci. Sports Exerc.; 40:2046-2054. 18981945 10.1249/MSS.0b013e318180bc6f

[b25] Ogoh S. Ainslie P. N. 2009a Cerebral blood flow during exercise: mechanisms of regulation. J. Appl. Physiol. (1985); 107:1370-1380. 19729591 10.1152/japplphysiol.00573.2009

[b26] Ogoh S. Ainslie P. N. 2009b Regulatory mechanisms of cerebral blood flow during exercise: new concepts. Exerc. Sport Sci. Rev.; 37:123-129. 19550203 10.1097/JES.0b013e3181aa64d7

[b27] Ogoh S. Brothers R. M. Barnes Q. Eubank W. L. Hawkins M. N. Purkayastha S. 2005a The effect of changes in cardiac output on middle cerebral artery mean blood velocity at rest and during exercise. J. Physiol.; 569:697-704. 16210355 10.1113/jphysiol.2005.095836 PMC1464249

[b28] Ogoh S. Dalsgaard M. K. Yoshiga C. C. Dawson E. A. Keller D. M. Raven P. B. 2005b Dynamic cerebral autoregulation during exhaustive exercise in humans. Am. J. Physiol. Heart Circ. Physiol.; 288:H1461-H1467. 15498819 10.1152/ajpheart.00948.2004

[b29] Ogoh S. Fadel P. J. Zhang R. Selmer C. Jans O. Secher N. H. 2005c Middle cerebral artery flow velocity and pulse pressure during dynamic exercise in humans. Am. J. Physiol. Heart Circ. Physiol.; 288:H1526-H1531. 15591094 10.1152/ajpheart.00979.2004

[b30] Sato K. Ogoh S. Hirasawa A. Oue A. Sadamoto T. 2011 The distribution of blood flow in the carotid and vertebral arteries during dynamic exercise in humans. J. Physiol.; 589:2847-2856. 21486813 10.1113/jphysiol.2010.204461 PMC3112559

[b31] Schreiber S. J. Gottschalk S. Weih M. Villringer A. Valdueza J. M. 2000 Assessment of blood flow velocity and diameter of the middle cerebral artery during the acetazolamide provocation test by use of transcranial Doppler sonography and MR imaging. AJNR Am. J. Neuroradiol.; 21:1207-1211. 10954270 PMC8174897

[b32] Serrador J. M. Picot P. A. Rutt B. K. Shoemaker J. K. Bondar R. L. 2000 MRI measures of middle cerebral artery diameter in conscious humans during simulated orthostasis. Stroke; 31:1672-1678. 10884472 10.1161/01.str.31.7.1672

[b33] Stroop J. R. 1935 Studies of interference in serial verbal reactions. J. Exp. Psychol.; 18:643-662.

[b34] Tomporowski P. D. 2003 Effects of acute bouts of exercise on cognition. Acta Psychol. (Amst); 112:297-324. 12595152 10.1016/s0001-6918(02)00134-8

[b35] Vasques P. E. Moraes H. Silveira H. Deslandes A. C. Laks J. 2011 Acute exercise improves cognition in the depressed elderly: the effect of dual‐tasks. Clinics (Sao Paulo); 66:1553-1557. 22179158 10.1590/S1807-59322011000900008 PMC3164403

[b36] Wingo J. E. Lafrenz A. J. Ganio M. S. Edwards G. L. Cureton K. J. 2005 Cardiovascular drift is related to reduced maximal oxygen uptake during heat stress. Med. Sci. Sports Exerc.; 37:248-255. 15692320 10.1249/01.mss.0000152731.33450.95

[b37] Zappe A. C. Uludag K. Oeltermann A. Ugurbil K. Logothetis N. K. 2008 The influence of moderate hypercapnia on neural activity in the anesthetized nonhuman primate. Cereb Cortex; 18:2666-2673. 18326521 10.1093/cercor/bhn023 PMC2567427

